# Supplementary material for: Splice-Junction-Based Mapping of Alternative Isoforms in the Human Proteome
Source: Cell Rep. Author manuscript; Available in PMC 2020 Jan 15. (PMC6961840; doi:10.1016/j.celrep.2019.11.026)
Supplement: 2 [file NIHMS1546469-supplement-2.zip › DF1/PXD006675/RightAtrium/RightAtrium_34_TPM3_AISEELDHALNDMTSGSQMLKLDK.pdf]

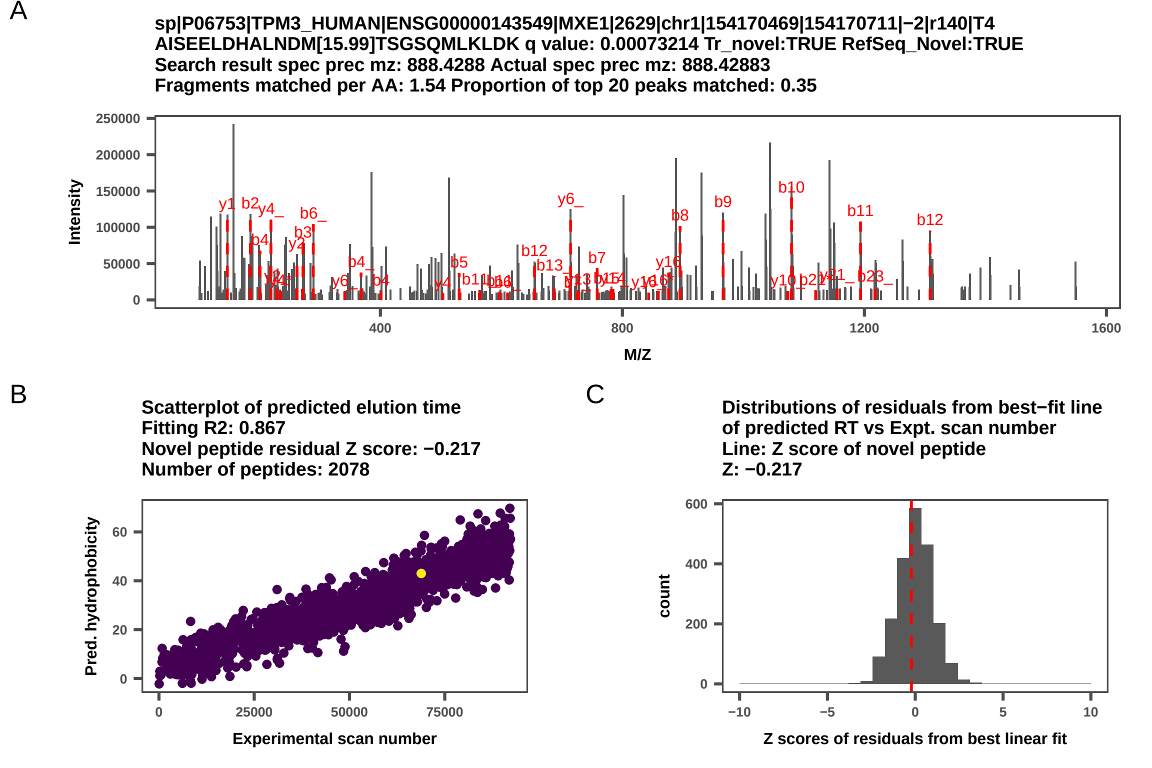
A
sp|P06753|TPM3_HUMAN|ENSG00000143549|MXE1|2629|chr1|154170469|154170711|−2|r140|T4
AISEELDHALNDM[15.99]TSGSQMLKLDK q value: 0.00073214 Tr_novel:TRUE RefSeq_Novel:TRUE
Search result spec prec mz: 888.4288 Actual spec prec mz: 888.42883
Fragments matched per AA: 1.54 Proportion of top 20 peaks matched: 0.35
Intensity
250000
200000
150000
100000
50000
0
y1
b2
y4_
b6_
b4
b3
y2
b4_
y6_
b4
y4
b5
b12
b13_
y6_
b7
y16
b8
b9
b10
y10_
b11
b12
400
800
1200
1600
M/Z
B
Scatterplot of predicted elution time
Fitting R2: 0.867
Novel peptide residual Z score: −0.217
Number of peptides: 2078
Pred. hydrophobicity
60
40
20
0
0
25000
50000
75000
Experimental scan number
C
Distributions of residuals from best−fit line
of predicted RT vs Expt. scan number
Line: Z score of novel peptide
Z: −0.217
count
600
400
200
0
−10
−5
0
5
10
Z scores of residuals from best linear fit
